# Older renal cell cancer patients experience increased rates of venous thromboembolic events: a retrospective cohort study of SEER-Medicare data

**DOI:** 10.1186/1471-2407-13-209

**Published:** 2013-04-27

**Authors:** Alexandra Connelly-Frost, Sumitra Shantakumar, Monica G Kobayashi, Haojie Li, Li Li

**Affiliations:** 1 Frost Consulting, Epidemiologic Research and Grant Writing, 1256 S. Kings Drive, Charlotte, NC, USA; 2 Department of Worldwide Epidemiology, Research and Development, GlaxoSmithKline, 5 Moore Drive, P.O. Box 13398, Research Triangle Park, NC, 17.2116, USA; 3 Department of Worldwide Epidemiology, Research and Development, GlaxoSmithKline, Collegeville, PA, USA

**Keywords:** Venous thromboembolism, Renal cell carcinoma, Incidence, Co-morbidity, Pulmonary embolism, Deep vein thrombosis

## Abstract

**Background:**

Venous thromboembolic co-morbidities can have a significant impact on treatment response, treatment options, quality of life, and ultimately, survival from cancer. The extent of venous thromboembolic co-morbidity among older renal cell cancer patients is poorly described in the literature. It is important to understand the scope of venous thromboembolic events, before and after diagnosis, in order to offer renal cell cancer patients optimal care and improved quality of life.

**Methods:**

The main goal of this study was to estimate and describe the incidence of venous thromboembolic events before and after renal cell cancer diagnosis. SEER-Medicare linked data (1991–2003) was utilized for this retrospective cohort analysis (n = 11,950) of older renal cell cancer patients (≥ 65 years). Incidence rates and proportions in addition to multivariable Cox proportional hazard and logistic regression models were utilized to describe the incidence and relative risk of venous thromboembolic events.

**Results:**

We observed that in the 12 months after diagnosis, 8.3% of renal cell cancer patients experienced a deep venous thrombosis, 2.4% experienced a pulmonary embolism, and 3.9% experienced other thromboembolic events. Nearly 70% of venous thromboembolic events occurred in the first 90 days after renal cell cancer diagnosis. Renal cell cancer patients were 2–4 times more likely to have a venous thromboembolic event in the 12 months after cancer diagnosis than non-cancer patients followed during the same time frame. Recent history of a venous event substantially increased the risk of that same event in the 12 months after diagnosis (HR = 5.2-18.8).

**Conclusion:**

Venous thromboembolic events are common and serious co-morbidities that should be closely monitored in older renal cell cancer patients, particularly during the first 3 months following diagnosis and among those with a recent history of a venous thromboembolic event.

## Background

Venous thromboembolic co-morbidities among cancer patients can have a significant impact on treatment response, treatment options, quality of life, and ultimately, survival from cancer [1-3]. Although it has been estimated that 1 in 200 cancer patients will experience venous thromboembolic events (VTEs), annually [4]; a detailed analysis of VTEs by cancer type is not available in the literature. A small body of evidence is developing for brain, breast, lung, ovarian, and pancreatic cancers, suggesting that the incidence of VTEs varies substantially by cancer subtype [2,5-14]. Estimates for these cancers range from as low as 0.4% up to 26.0%, depending on the cancer type, study population, and the length of follow-up [13]. Because each cancer is a distinct disease, it is important to carefully characterize this co-morbidity by cancer subtype.

The extent of venous thromboembolic co-morbidity among older renal cell cancer (RCC) patients is poorly described in the literature: timing of VTEs has not been thoroughly investigated, cofactors have not been adequately considered, VTE subgroups have not been evaluated, older subgroups have not been studied, and the broader diagnosis category of kidney cancer has been used instead of the more specific diagnosis of RCC. It is important to understand the scope of VTEs, before and after diagnosis, in order to offer RCC patients optimal care and improved quality of life.

The primary objectives of this study were 1) to estimate the incidence of VTEs before RCC diagnosis and during various time periods after RCC diagnosis 2) to produce adjusted relative risk estimates of VTEs for RCC patients with and without a cardiovascular disease or VTE history and 3) to compare risk of VTEs for RCC patients versus age-matched non-cancer individuals.

## Methods

### Study population

SEER-Medicare data is a linkage of U.S. cancer registry data with Medicare claims data. This database combines two large, population-based, geographically diverse U.S. data sources, providing detailed information about elderly persons (≥65 years) with and without cancer. Data from 1991–2003 were utilized for this retrospective cohort analysis. Patients 65 years of age and over who were diagnosed with RCC and had at least 24 months of continuous non-HMO Medicare coverage (Parts A and B) before diagnosis and 1 to 12 months after diagnosis were included in the cancer cohort. Duration of patient follow-up after diagnosis (maximum 12 months) was the number of months until the patient died or lost Medicare coverage. If neither of these events occurred before the end of the planned follow-up time after diagnosis, the patient was followed for the full 12 months. Non-cancer patients were frequency-matched by age to cancer patients at a ratio of 1:1. VTEs of interest were deep vein thrombosis (DVT), pulmonary embolism (PE), and other thromboembolic events (OTE). ICD-9 diagnosis codes were used to identify VTEs and ICD-O-3 codes were used to identify RCC patients. DVT was captured using ICD-9 codes of 451.1 (451.11, 451.19) 451.2, 451.81, 451.83, 451.84, 453.1, 453.2, 453.4 (453.40, 453.41, 453.42) 453.8, and 453.9; PE was captured using ICD-9 codes of 415.1 and 415.19. OTEs were captured using ICD-9 codes of 362.35, 362.36, 437.6, 451.0, 451.82, 451.89, 451.9, 453.0, 453.3, and 452. Renal Cell Carcinoma (RCC) was captured using ICD-O-3 of C649 excluding histologies 8050–8130 (inclusive) and any leukemias and lymphomas of the renal pelvis.

### Statistical analysis

#### RCC patients

Incidence rates of each VTE a) in the 12 months before diagnosis and b) in the 12 months after diagnosis were described by age, race, sex, stage at diagnosis, and year of diagnosis. The numerator is the number of events that occurred over the respective 12-month period and the denominator is the person-years at risk. Events in the 12 months after diagnosis were further described as the proportion of cases with a first event in discrete time intervals of follow-up time (0–90 days, 91–180 days, 181–270 days, and 271–365 days). The numerator of each incidence proportion is the number of persons with their first event of interest during that time period only, while the denominator represents the persons who were alive and free of events at the beginning of the period.

The Cox proportional hazard model was used to build predictive models to identify important risk factors for each VTE of interest among RCC patients. Potential risk factors included in the initial (full) model were as follows: age at diagnosis, race, sex, diabetes, hypercholesterolemia, atherosclerosis, varicose veins, recent high-risk surgical procedure, central venous catheter, sickle cell anemia, kidney disease, stage at diagnosis, chemotherapy, immunotherapy, hormone therapy, surgery of primary site, history of cancer, and recent history of VTE. Risk factors with a multivariable p-value <0.1 were retained in the final multivariable predictive model.

Relative incidence rates of VTEs in RCC patients with a recent history of cardiovascular event (CVD) or VTE event (12 months before diagnosis) versus RCC patients without such a recent history were calculated using the Cox proportional hazard models. History of CVD event was defined as any of the following events in the 12 months before RCC diagnosis: myocardial infarction, ischemic stroke, congestive heart failure, angina, or TIA. The first VTE was counted for each patient from time of diagnosis up to 12 months after diagnosis. Potential confounders and effect measure modifiers, identified through ICD-9 diagnosis and procedure codes, were as follows: age at diagnosis, race, sex, diabetes, hypercholesterolemia, atherosclerosis, varicose veins, recent high-risk surgical procedure (cardiac or vascular surgeries in the year after RCC diagnosis), central venous catheter, sickle cell anemia, kidney disease, stage at diagnosis, chemotherapy, nephrectomy, immunotherapy, hormone therapy, history of cancer, recent history of CVD event, and recent history of VTE.

#### RCC vs. Non-cancer patients

A matched-cohort design was utilized to compare rates of VTEs among RCC and similar non-cancer patients. Multivariable logistic regression analysis was used to evaluate the relative risk of VTEs in RCC patients (12 months before diagnosis) versus non-cancer cases (12 months before index date). Relative incidence rate of VTEs in RCC patients (12 months after diagnosis) versus non-cancer cases (12 months after index date) was calculated using multivariable Cox proportional hazard models. Potential confounders and effect measure modifiers (refer to previous section) were assessed and all models were adjusted for age to account for the age-matched design. Sas 9.1 was used to perform all analyses.

This data analysis was approved by our protocol review forum for scientific merit. Because we analyzed secondary, observational data which was anonymized and distributed by the National Cancer Institute (SEER-Medicare data), this project was not reviewed separately by an ethics committee. Representatives from the NCI and SEER reviewed this project for potential confidentiality concerns prior to releasing the study data.

## Results

The study population for the first series of analyses consisted of 11,950 RCC patients 65 years of age and older (median age = 75 years). Eighty-six percent of the population was white, 8% of the population was black, and 6% was another race. Fifty-nine percent of RCC patients were male. The distribution of cases by stage was as follows: 52% localized, 19% regional, 21% distant, and 8% unstaged. The non-cancer comparison cohort was similar in its distribution of age and race; however, the proportion of males in the non-cancer population (37%) was lower than in the RCC cohort (59%).

### RCC patients

**Figure 1:**
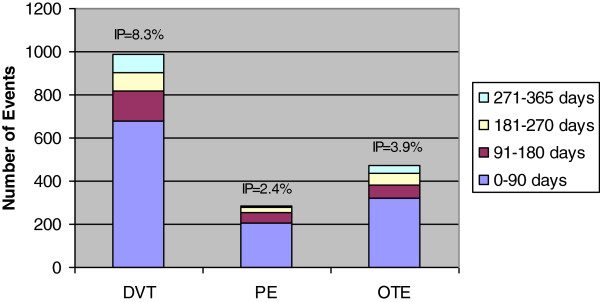
**VTEs in discrete time intervals and incidence proportions**^**a **^**in the 12 months after RCC diagnosis**^**b**^**. **^a^Incidence proportions = IP = 1-year overall incidence proportion defined as number of events divided by the beginning population at risk. ^b^Results from SEER-Medicare Data (1991–2003).

Incidence rate of VTEs among older RCC patients was 2.1-3.8 times higher during the 12-month period after RCC diagnosis than the 12-month period prior to cancer diagnosis (Table 1). Of all VTEs that occurred after diagnosis, DVTs occurred at the highest rate (108/ 1,000 person-years). The number of VTEs experienced in discrete time periods after diagnosis and one-year incidence proportions for the 12 month period among RCC patients are displayed in Figure 1. Among RCC patients, 8.3% experienced a DVT, 2.4% experienced a PE, and 3.9% experienced OTEs in the 12 months after diagnosis (Figure 1). Regardless of type of VTE, nearly 70% of VTEs occurred in the first 90 days after RCC diagnosis: DVT: 68.6% (679/990), PE: 71.3% (204/286), and OTE: 67.7% (319/471).

**Table 1:** **Unadjusted incidence rates of VTEs among older RCC patients, before and after RCC diagnosis SEER-Medicare Data (1991–2003)**^**a**^

**n = 11,950 RCC Patients**	**Incidence 12 months before RCC diagnosis**	**Incidence 12 months after RCC diagnosis**
DVT^**a**^		
n/person-years^b^	380/11,785	990/9,150
Rate/1,000 ^**c**^	32.2 (29.1-35.7)	108.2 (101.6-115.2)
*Rate ratio (after vs. before)*^*d*^	--	*3.4 (3.0-3.8)*
PE^**a**^		
n/person-years^b^	95/11,912	286/9,536
Rate/1,000 ^**c**^	8.0 (6.5-9.8)	30.0 (26.6-33.7)
*Rate ratio (after vs. before)*^*d*^	--	*3.8 (3.0-4.7)*
OTE^**a**^		
n/person-years^b^	280/11,813	462/9,434
Rate/1,000 ^**c**^	23.7 (21.1-26.7)	49.0 (44.6-53.7)
*Rate ratio (after vs. before)*^*d*^	--	*2.1 (1.8-2.4)*

^a^VTE = venous thromboembolic events; RCC = renal cell cancer; DVT = Deep Vein Thrombosis; PE = Pulmonary Embolism; OTE = Other thromboembolic event. OTE category includes the following diagnoses: central retinal vein occlusion, venous tributary (branch) occlusion, Nonpyogenic thrombosis of intracranial venous sinus, phlebitis/thrombophlebitis of superficial vessels of lower extremities, phlebitis/thrombophlebitis of superficial veins of upper extremities, phlebitis/thrombophlebitis of other sites, gout with other specified manifestations, Budd-Chiari syndrome, and venous embolism/thrombosis of renal vein.

^b^n = number of VTE events; p-y = person-years.

^c^Rates are per 1,000 person-years and are unadjusted. Age adjustment is unnecessary as these rates are intentionally representative of the older subpopulation (ages 65+) of RCC patients. Only first VTE counted in rate estimates.

^d^Rate ratios are unadjusted.

Unadjusted analyses revealed that RCC patients with a recent history of a VTE had substantially higher rates of that specific VTE after RCC diagnosis than those without history of that VTE (Table 2). Patients of advanced or regional RCC stage had higher rates of VTEs after diagnosis compared to patients with localized disease (Table 2). Patients who received immunotherapy were also more likely to experience all three VTEs, particularly DVT. Patients who were treated with nephrectomy were less likely to experience a DVT or PE after RCC diagnosis (Table 2).

**Table 2:** Incidence rates of VTE among older RCC patients in the 12 months after cancer diagnosis

	**DVT**^**a**^		**PE**^**a**^		**OTE**^**a**^	
**n = 11,950 RCC patients**	**N / P-Y**^**b**^	**Rate/1,000**^**c **^**(95% CI)**	**N / P-Y**^**b**^	**Rate/1,000**^**c **^**(95% CI)**	**N / P-Y**^**b**^	**Rate/1,000**^**c **^**(95% CI)**
Age						
65-69	144/1,593	90.4 (76.2-106.4)	39/1,657	23.5 (16.7-32.2)	59/1,638	36.0 (27.4-46.5)
70-74	306/2,883	106.2 (94.6-118.7)	84/3,011	27.9 (22.3-34.5)	170/2,965	57.4 (49.1-66.6)
75-79	270/2,436	110.8 (98.0-124.4)	84/2,527	33.3 (26.5-41.2)	121/2,512	48.2 (40.0-57.6)
80-84	175/1,479	118.3 (101.4-137.2)	60/1,546	38.8 (29.6-50.0)	75/1,539	48.7 (38.3-61.1)
85+	95/759	125.2 (101.3-153.1)	19/795	23.9 (14.4-37.3)	37/781	47.4 (33.4-65.3)
Race						
Black	104/709	146.6 (119.8-177.1)	28/753	37.2 (24.7-53.8)	35/751	46.6 (32.4-64.8)
White	838/7,908	106.0 (98.9-113.4)	248/8,227	30.1 (26.5-34.1)	405/8135	49.8 (45.1-54.9)
Other	46/520	88.5 (64.8-188.1)	10/542	18.5 (8.9-34.0)	21/534	39.3 (24.3-60.1)
Sex						
Female	440/3,681	119.5 (108.6-131.2)	129/3,850	33.5 (28.0-39.8)	202/3,809	53.0 (46.0-60.9)
Male	550/5,469	100.6 (92.3-109.3)	157/5,686	27.6 (23.5-32.3)	260/5,625	46.2 (40.8-52.2)
History of VTE^d^						
Yes	93/86	1086.7 (877.1-1,331.3)	24/25	974.3 (624.3-1,449.7)	37/77	482.8 (339.9-665.4)
No	897/9,065	99.0 (92.6-105.7)	262/9,511	27.6 (24.3-31.1)	425/9,357	45.4 (41.2-50.0)
History of CVD^e^						
Yes	181/1,400	129.3 (111.1-149.5)	54/1,465	36.9 (27.7-48.1)	84/1,451	57.9 (46.2-71.7)
No	809/7,750	104.4 (97.3-111.8)	232/8,071	28.7 (25.2-32.7)	378/7,983	47.4 (42.7-52.4)
Disease stage						
Localized	374/5,628	66.5 (59.9-73.5)	133/5,757	23.1 (19.3-27.4)	190/5,734	33.1 (28.6-38.2)
Regional	290/1,749	165.8 (147.3-186.1)	62/1,899	32.7 (25.0-41.9)	154/1,837	83.9 (71.1-98.2)
Distant	266/1,161	229.2 (202.5-258.5)	69/1,245	55.4 (43.1-70.2)	90/1,230	73.2 (58.8-89.9)
Immunotherapy^f^						
Yes	78/376	207.3 (163.8-258.6)	17/407	41.7 (24.3-66.8)	27/398	67.9 (44.7-98.7)
No	912/8,774	103.9 (97.3-110.9)	269/9,128	29.5 (26.0-33.2)	435/9,036	48.1 (43.7-52.9)
Nephrectomy						
Yes	665/6,963	95.5 (88.4-103.1)	204/7,250	28.1 (24.4-32.3)	341/7,164	47.6 (42.7-52.9)
No	325/2,188	148.6 (132.9-165.6)	82/2,286	35.9 (28.5-44.5)	121/2270	53.3 (44.2-63.7)

^a^VTE = venous thromboembolic events; RCC = renal cell cancer; DVT = Deep Vein Thrombosis; PE = Pulmonary Embolism; OTE = Other thromboembolic event. OTE category includes the following diagnoses: central retinal vein occlusion, venous tributary (branch) occlusion, Nonpyogenic thrombosis of intracranial venous sinus, phlebitis/thrombophlebitis of superficial vessels of lower extremities, phlebitis/thrombophlebitis of superficial veins of upper extremities, phlebitis/thrombophlebitis of other sites, gout with other specified manifestations, Budd-Chiari syndrome, and venous embolism/thrombosis of renal vein.

^b^N = number of VTE events; P-Y = person-years.

^c^Rates are per 1,000 person-years and are unadjusted. Age adjustment is unnecessary as these rates are intentionally representative of the older subpopulation (ages 65+) of RCC patients. Only first VTE counted in rate estimates.

^d^History of VTE of interest in the 12 months before RCC diagnosis.

^e^History of CVD is defined as a history of any of the following events in the 12 months before RCC diagnosis: myocardial infarction, ischemic stroke, onset congestive heart failure, angina, or TIA.

^f^Immunotherapy in our data (1991–2003) predominantly included Interferon alpha and IL-2 therapies.

The strongest predictor of increased risk of a VTE in the 12 months after RCC diagnosis was a recent history of that particular VTE event (Table 3; HR range = 5.4-20.1). Stage at diagnosis, atherosclerosis, and kidney disease were also significantly associated with an increased risk of VTE. Predictors associated with decreased risk of VTE were prior use of central venous catheter and recent experience of high-risk surgery.

**Table 3:** **Important Predictors of VTEs in the 12 months after RCC diagnosis, SEER-Medicare Data (1991–2003)**^**a**^

**n = 11,950 RCC patients**	**DVT**^**b **^**(n = 990)**	**P-Value**	**PE**^**b **^**(n = 286)**	**P-Value**	**OTE**^**b **^**(n = 470)**	**P-Value**
	**HR (95% CI)**		**HR (95% CI)**		**HR (95% CI)**	
Male sex	0.8 (0.7-0.9)	<0.001	0.8 (0.6- 1.0)	0.041	--	--
Atherosclerosis	2.0 (1.7-2.3)	<0.001	2.5 (2.0-3.2)	<0.001	1.7 (1.4-2.1)	<0.001
Diabetes	1.2 (1.1-1.4)	0.004	--	--	--	--
Hypercholesterolemia	--	--	--	--	1.3 (1.1-1.6)	0.012
Kidney disease	1.9 (1.6-2.1)	<0.001	1.6 (1.2- 2.0)	<0.001	1.4 (1.2-1.7)	0.001
Varicose veins	2.2 (1.6-3.1)	<0.001	--	--	--	--
History of cancer diagnosis	--	--	1.5 (1.0- 2.2)	0.033	--	--
History of VTE^c^	5.4 (4.4-6.4)	<0.001	20.1 (13.8-29.2)	<0.001	7.6 (5.9-9.9)	<0.001
Chemotherapy	1.8 (1.4-2.2)	<0.001	--	--	1.4 (1.0-2.0)	0.048
Central venous catheter^d^	0.4 (0.3-0.4)	<0.001	0.3 (0.2-0.5)	<0.001	0.5 (0.4-0.7)	<0.001
High-risk surgery^e^	0.4 (0.3-0.6)	<0.001	0.5 (0.3-0.8)	0.003	0.5 (0.4-0.7)	<0.001
Stage						
Regional versus localized	2.5 (2.2-2.9)	<0.001	1.6 (1.2-2.1)	0.002	2.6 (2.1-3.2)	<0.001
Distant versus localized	2.6 (2.2-3.0)	<0.001	1.9 (1.4-2.5)	<0.001	1.7 (1.3-2.2)	<0.001

^a^All models adjusted for age and race (age and race were not statistically significant predictors of any VTEs).

^b^DVT = Deep Vein Thrombosis; PE = Pulmonary Embolism; OTE = Other thromboembolic event. OTE category includes the following diagnoses: central retinal vein occlusion, venous tributary (branch) occlusion, Nonpyogenic thrombosis of intracranial venous sinus, phlebitis/thrombophlebitis of superficial vessels of lower extremities, phlebitis/thrombophlebitis of superficial veins of upper extremities, phlebitis/thrombophlebitis of other sites, gout with other specified manifestations, Budd-Chiari syndrome, and venous embolism/thrombosis of renal vein.

^c^History of VTE in the 12 months before RCC diagnosis.

^d^Central venous catheter (CVC) in the 12 months after diagnosis. CVCs that occurred less than 30 days before a TE event were excluded.

^e^High-risk surgery = cardiac or vascular surgeries in the year after RCC diagnosis. Procedures that happened less than 30 days before a TE event were excluded because of the nature of Medicare claims (Date ranges are used for procedures as we wanted to make sure that the procedure was not part of the treatment for the VTE outcome of interest).

Multivariate modeling was conducted to more closely evaluate the association between CVD history, VTE history, and incidence of VTEs after RCC diagnosis. RCC patients with a recent history of a CVD event (MI, IS, congestive heart failure, angina, TIA) were no more likely to have a VTE after RCC diagnosis than those without CVD event history (results not shown). On the other hand, a history of the specific VTE substantially increased the risk of that venous event in the 12 months after diagnosis (HR = 5.2-18.8) (Table 4). The association between recent history of DVT and DVT occurrence after diagnosis was modified by the presence of kidney disease; among patients who did not have kidney disease, the association was almost twice as strong (HR = 6.8, 95% CI = 5.4-8.7) as among those with kidney disease (HR = 3.8, 95% CI = 2.9-5.1). The association between recent history of PE and PE occurrence after diagnosis was the strongest (HR = 18.9, 95% CI = 13.0-27.5) and was not modified by any covariates. The association between having a recent history of OTE and OTE occurrence after diagnosis was modified by the presence of kidney disease and sex. Among patients who did not have kidney disease, the association between history of OTE and OTE after diagnosis was nearly three times as strong as among those with kidney disease (did not have kidney disease: HR = 10.2, 95% CI 7.5-14.0; had kidney disease: HR = 3.9, 95% CI = 2.5-6.4). The same association was also much stronger among men (HR = 11.5, 95% CI = 8.4-15.7) than women (HR = 3.4, 95% CI = 2.1-5.6).

**Table 4:** **Relative risk of VTE after RCC diagnosis, by recent VTE history**^**a**^

**n = 11,950**	**DVT**^**b**^	**PE**^**b**^	**OTE**^**b**^
	**HR (95% CI)**	**HR (95% CI)**	**HR (95% CI)**
***Overall hazard ratios***	*5.2 ( 4.4-6.3)*	*18.8 (13.0-27.5)*	*7.1 (5.5-9.2)*
***P-Value***^***c***^	*<0.001*	*<0.001*	*<0.001*
**Effect measure modifiers**			
Kidney disease			
Yes	3.8 (2.9-5.1)	ns	3.9 (2.5- 6.4)
No	6.8 (5.4-8.7)	ns	10.2 (7.5-14.0)
P-Value	0.003	0.432	<0.001
Sex			
F	ns	ns	3.4 (2.1- 5.6)
M	ns	ns	11.5 (8.4-15.7)
P-Value	0.818	0.239	<0.001

^a^All models adjusted for atherosclerosis, kidney disease, and sex. Hazard ratios compared the risk of a VTE in the 12 months after diagnosis for those with versus without a recent history of the specific VTE of interest. Results from SEER-Medicare Data (1991–2003).

^b^DVT = Deep Vein Thrombosis; PE = Pulmonary Embolism; OTE = Other thromboembolic event. OTE category includes the following diagnoses: central retinal vein occlusion, venous tributary (branch) occlusion, Nonpyogenic thrombosis of intracranial venous sinus, phlebitis/thrombophlebitis of superficial vessels of lower extremities, phlebitis/thrombophlebitis of superficial veins of upper extremities, phlebitis/thrombophlebitis of other sites, gout with other specified manifestations, Budd-Chiari syndrome, and venous embolism/thrombosis of renal vein.

^c^P-Value for the difference between stratum specific estimates.

ns = no statistically significant difference between strata.

### RCC patients compared to an age-matched, non-cancer population

Older RCC patients were 1.5-1.8 times more likely to have experienced a VTE in the recent past (i.e. 12 month prior to RCC diagnosis) than non-cancer individuals (Table 5). This was especially evident among those without a recent history of a CVD event. The association between RCC and having a VTE during 12 months after diagnosis was even stronger: RCC patients are 2.4-4.3 times more likely to experience a VTE event than age-matched non-cancer individuals during the same time frame (Table 5). Several factors modified these associations. The general pattern was that among patients without strong CVD risk factors (e.g. history of CVD events and diabetes), the association between RCC and VTEs was stronger than among those with a CVD risk factor.

**Table 5:** **Relative risk of VTE before and after diagnosis (or index date): RCC versus non-cancer cohort**^**a**^

		**DVT**^**b**^		**PE**^**b**^		**OTE**^**b**^	
		**Before**	**After**	**Before**	**After**	**Before**	**After**
		**OR (95% CI)**	**HR (95% CI)**	**OR (95% CI)**	**HR (95% CI)**	**OR (95% CI)**	**HR (95% CI)**
***Overall***		*1.6 (1.3-1.9)*	*3.6 (3.1-4.1)*	*1.8 (1.3-2.6)*	*4.3 (3.2-5.7)*	*1.5 (1.2-1.8)*	*2.4 (2.0-2.8)*
***P-value***^***c***^		*<0.001*	*<0.001*	*<0.001*	*<0.001*	*<0.001*	*<0.001*
**Effect measure modifiers**							
Atherosclerosis	Yes	ns	2.0 (1.5-2.6)	ns	ns	ns	ns
	No	ns	4.1 (3.5-4.9)	ns	ns	ns	ns
	P-value^c^	0.384	<0.001	0.112	0.275	0.364	0.100
Central venous catheter^d^	Yes	ns	ns	ns	0.7 (0.1-6.0)	ns	ns
	No	ns	ns	ns	4.9 (3.7-6.5)	ns	ns
	P-value ^c^	0.134	0.081	0.977	0.161	0.440	0.882
Diabetes	Yes	1.1 (0.8-1.5)	2.4 (1.9-3.1)	ns	ns	ns	ns
	No	1.8 (1.4-2.2)	4.2 (3.6-5.1)	ns	ns	ns	ns
	P-value ^c^	0.003	<0.001	0.280	0.332	0.181	0.108
High-risk surgery^e^	Yes	ns	1.4 (0.7-2.8)	ns	1.2 (0.4-3.7)	ns	ns
	No	ns	3.8 (3.3-4.4)	ns	4.7 (3.5-6.3)	ns	ns
	P-value ^c^	0.129	<0.001	0.698	0.030	0.714	0.387
History of CVD^f^	Yes	1.1 (0.8-1.5)	2.0 (1.6-2.6)	ns	2.5 (1.5-4.0)	1.0 (0.7-1.4)	1.6 (1.2-2.2)
	No	1.8 (1.4-2.2)	4.7 (3.9-5.6)	ns	5.2 (3.6-7.4)	1.7 (1.3-2.1)	2.7 (2.2-3.3)
	P-value ^c^	0.002	<0.001	0.366	0.005	0.001	<0.001
Kidney disease	Yes	ns	1.8 (1.3-2.5)	ns	1.3 (0.7-2.3)	ns	1.3 (0.8-2.2)
	No	ns	4.0 (3.4-4.6)	ns	5.2 (3.8-7.2)	ns	2.5 (2.1-3.0)
	P-value ^c^	0.292	<0.001	0.940	<0.001	0.343	0.014

^a^All models adjusted for age at index date (matching factor), sex, kidney disease and stratified by important effect measure modifiers. Odds ratios compared risk of a VTE in the 12 months before diagnosis (or index date) and hazard ratios compared risk of VTE in the 12 months after diagnosis (or index date). Results from SEER-Medicare Data (1991–2003). RCC Patients (n = 11,950); Non-Cancer Control Group (n = 11,918).

^b^DVT = deep vein thrombosis, PE = pulmonary embolism, OTE = other thrombolic event. OTE includes: central retinal vein occlusion, venous tributary (branch) occlusion, nonpyogenic thrombosis of intracranial venous sinus, phlebitis/thrombophlebitis of superficial vessels of lower extremities, phlebitis/thrombophlebitis of superficial veins of upper extremities, phlebitis/thrombophlebitis of other sites, gout with other specified manifestations, Budd-Chiari syndrome, venous embolism/thrombosis of renal vein and portal vein thrombosis.

^c^P-Value for the difference between stratum specific estimates.

^d^Central venous catheter (CVC) in the 12 months after diagnosis. CVCs that occurred less than 30 days before a TE event were excluded.

^e^High-risk surgery = cardiac or vascular surgeries in the year after index date or RCC diagnosis. Procedures that happened less than 30 days before a TE event were excluded because of the nature of Medicare claims (Date ranges, rather than exact dates, are used for procedures and we wanted to make sure that the procedure was not part of the treatment for the TE outcome of interest.).

^f^History of CVD = any of the following CVD events in the year before the analysis period: MI, IS, onset congestive heart failure, angina, or TIA (analysis period = 12 months before diagnosis or index date or 12 months after diagnosis or index date).

ns = no statistically significant difference between strata.

## Discussion

To compare our results to those in the literature, we combined our DVT and PE incidence and incidence density estimates to determine an overall VTE incidence, commonly reported by others. Our combined (DVT + PE), one-year, post RCC incidence was 10.7% and incidence density rate was 127/1,000 p-y. These estimates are higher than what has been reported in the literature thus far [2,6-8,10-12].

Our incidence results, however, are difficult to compare to current literature because of method and study population differences. Our analysis followed each patient for a defined period after diagnosis or death to evaluate incidence over the full 12-month period after diagnosis; however, many of the studies calculated an incidence proportion (percent) based on whether the patient had a diagnosis of VTE during their initial hospitalization [7,10] or during a randomly selected hospitalization [8]. Their estimates of VTE among kidney cancer patients ranged from 0.8-7.6% per hospitalization. These are not comparable to our estimate(s) because only one hospitalization has been evaluated per patient. Stein et al. took an approach similar to ours in that they looked at all hospitalizations for each patient over a period of time; however, they also presented their results per hospitalization instead of per person or person-years (VTE incidence of 20/1,000 hospitalizations) [12]. It is unclear what the average length of follow-up was in their study and their study population was also substantially younger than ours, with ages ranging from 40–79 compared to our study age range of 65–101, with a median age of 75.

A few other studies presented cumulative incidence and incidence density results for kidney cancer patients; however, their study populations were vastly different from ours. Blom et al. found that 1.3% of kidney cancer patients (and 12.6/1,000 p-y) had a VTE in the 6 months after cancer diagnosis; however, researchers only included patients who visited an anticoagulant clinic, not patients who were hospitalized for treatment [6]. Their estimate is significantly lower than ours (127/1,000 p-y) and likely an underestimate of the rate in the general kidney cancer population because sicker individuals, who were unable to visit this ambulatory clinic, were not given the opportunity to present as a VTE case in their study population. They also had a younger population (median age = 64) than ours. Chew et al. performed their analysis very similarly to ours; however, they excluded all patients with a past diagnosis of VTE [2]. Their estimates of post kidney cancer VTE incidence (2-yr cumulative incidence: local: 1.3%, regional: 3.8, remote 3.5% and 2-yr incidence densities of 13, 37, and 60 per 1,000 p-y) are likely lower than ours because they have excluded the highest risk group of patients from their analysis. Sallah et al. found that 22.6% of kidney cancer patients developed VTE over an average of 26 months [11]. Their estimate of cumulative incidence was higher than ours; however, their sample size was very small (n = 31), their study population was much younger (median age = 60 yrs), and their average period of follow-up was twice as long as ours.

Our results indicate that the risk of VTE is highest in the first 90 days after RCC diagnosis. Blom et al. also found the risk of VTE particularly high during the first few months after cancer diagnosis; however, they could draw no conclusion about kidney cancer, in particular, due to limited sample size for that cancer (n = 8) [15]. The risk of VTE was significantly higher among distant metastasized kidney cancers in the Blom study, as well [15]. This is similar to our finding that regional and distant RCC patients were at increased risk of VTE compared to local RCC cases.

The major risk factors for venous thromboembolisms among cancer patients reported in the literature are increased age, female sex, African American race, renal disease, infection, pulmonary disease, obesity, arterial thromboembolism, inherited prothrombotic mutations, prior history of VTE, performance status, advanced stage cancer, major surgery, hospitalization, chemotherapy, hormone therapy, anti-angiogenic agents, erythropoiesis-stimulating agents, transfusions, and central venous catheters [4,8,13,16,17]. In our predictive model, many of these risk factors proved to be predictors of VTEs in the 12 months after RCC diagnosis. Unadjusted results presented in Table 2 suggested that immunotherapy might be an important predictor of VTEs in our data; however, after adjustment for stage and other important risk factors (Table 3), immunotherapy was not a statistically significant predictor of VTEs.

A few interesting differences are worth discussion. Atherosclerosis was a strong predictor for DVT, PE and OTE events. This condition is not generally mentioned as a risk factor for VTE among cancer patients; however, cardiovascular literature has suggested a link between these two conditions [18-21]. Another interesting result was that central venous catheter (CVC) and high-risk surgery decreased the risk VTE in our data. Decreased risk in this subgroup of patients is likely due to the close monitoring and prophylactic treatment for venous thromboses in surgical and catheterization situations.

An important component of our analysis was the evaluation of VTE history as a risk factor for future VTE events. Our results suggest that VTE history is the most important factor to consider in evaluating risk of future VTE in RCC patients. There are no other studies in the current literature that quantify the association between VTE history and RCC; however, this result is consistent with broader studies of VTE among cancer patients [4,17].

Finally, our analysis compared the risk of VTE events in RCC versus non-cancer patients both before and after RCC diagnosis. Our study found that RCC patients were 1.5-1.9 times more likely to have experienced a VTE in the recent past (i.e. 12 month before RCC diagnosis) than non-cancer individuals. White et al. reported a similar result: the standardized incidence ratio of observed versus expected RCC patients was 2.5 among those with a history of VTE [22]. No other published studies had sufficient numbers to address the relation between VTE and subsequent RCC diagnosis. These results support the common theory that VTE could be a risk marker for an ensuing cancer diagnosis [4,23,24].

Our study found that RCC patients were also 3.6 times more likely to experience a DVT and 4.3 times more likely to experience a PE event in the 12 months after RCC diagnosis than age-matched non-cancer individuals during the same time frame. Stein et al. reported a slightly weaker association: RCC cancer patients were 2.0 times more likely to have a DVT and 1.7 times more likely to have a PE than non-cancer patients [12]. The study population was significantly younger in the Stein study and they did not adjust for confounders or evaluate effect modifiers. Blom et al. also compared VTE incidence among kidney cancer patients versus non-cancer patients (OR = 6.2, 95% CI 0.8-46.5); however, there were only 8 kidney cancer cases in their case–control study [15].

There are several strengths of note for this study. To our knowledge, this is the first study to examine VTEs among older RCC patients. In this analysis we were able to focus on RCC patients in particular, rather than kidney cancer patients overall, because of the availability of detailed histological information in SEER. The RCC patient cohort was large (n = 11,950) allowing us to produce precise effect measure estimates, even after stratification. Unlike many published studies, which combined TE events into one outcome group, we examined three individual venous outcome groups (DVT, PE and OTEs) based on ICD-9 diagnostic codes. The wealth of the data in the SEER-Medicare database allowed us to quantify the occurrence of TE events before RCC diagnosis and during various time periods after RCC diagnosis, and to make comparisons between RCC patients and age-matched non-cancer individuals. Furthermore, we were able to adjust for and/or stratify by important covariates in our analysis. All estimates for incidence of VTEs among RCCs in previous literature were generated from studies that looked at multiple cancers and presented unadjusted incidence estimates for specific cancers, usually per hospitalization. No studies performed multivariate analyses on RCC patients nor did they investigate the timing of VTE events among RCC patients.

As in any study, limitations were present. The results based on this older cohort (i.e., 65 years or older) are generalizable only to those of the same age group. Also, information on some behavioral risk factors such as smoking, sedentary lifestyle, immobility, and CVD family history was unavailable. Oral prescription information was also unavailable, precluding the evaluation of anti-platelet therapy or anti-coagulant use. Finally, we had no access to information about potential predictive biomarkers such as elevated platelet or leukocyte counts, tissue factor, soluble p-Selectin, D-dimer, factor V Leiden, and prothrombin 20210A mutations [13,15,25].

## Conclusion

This is the first study to perform an in depth analysis of VTEs among RCC patients. Our results indicate that RCC patients are at increased risk of VTEs after cancer diagnosis and that patients diagnosed with RCC are more likely to have had a VTE within 12 months before their RCC diagnosis. VTEs are common and serious co-morbidities that should be closely monitored in older RCC patients, particularly during the first three months after diagnosis and among those with a recent history of a VTE.

## Abbreviations

VTE: Venous thromboembolic event; RCC: Renal cell cancer; CVD: Cardiovascular disease; PE: Pulmonary embolism; DVT: Deep vein thrombosis; OTE: Other thromboembolic event; SEER: Surveillance epidemiology and end results

## Competing interests

At the time of manuscript preparation, all authors were employed by or served as a consultant for GlaxoSmithKline.

## Authors’ contributions

AC participated in the conception and design of the study, analysis and interpretation of data, drafting of the manuscript, and critical revision of the manuscript for important intellectual content. SS participated in the conception and design of the study, acquisition of data, analysis and interpretation of data, and critical revision of the manuscript for important intellectual content. HL participated in the concept and design of the study, analysis and interpretation of data, and critical revision of the manuscript for important intellectual content. LL and MK participated in management and statistical analysis of data. All authors read and approved the final manuscript.

## Pre-publication history

The pre-publication history for this paper can be accessed here:

http://www.biomedcentral.com/1471-2407/13/209/prepub

## References

[B1] Sorensen HT Mellemkjaer L Olsen JH Baron JA Prognosis of cancers associated with venous thromboembolism N Engl J Med 2000 343 1846 1850 10.1056/NEJM200012213432504 11117976

[B2] Chew HK Wun T Harvey D Zhou H White RH Incidence of venous thromboembolism and its effect on survival among patients with common cancers Arch Intern Med 2006 166 458 464 16505267 10.1001/archinte.166.4.458

[B3] Khorana AA Francis CW Culakova E Kuderer NM Lyman GH Thromboembolism is a leading cause of death in cancer patients receiving outpatient chemotherapy J Thromb Haemost 2007 5 632 634 10.1111/j.1538-7836.2007.02374.x 17319909

[B4] Lee AY Levine MN Venous thromboembolism and cancer: risks and outcomes Circulation 2003 107 I17 I21 12814981 10.1161/01.CIR.0000078466.72504.AC

[B5] Agnelli G Bolis G Capussotti L Scarpa RM Tonelli F Bonizzoni E A clinical outcome-based prospective study on venous thromboembolism after cancer surgery: the @RISTOS project Ann Surg 2006 243 89 95 10.1097/01.sla.0000193959.44677.48 16371741 PMC1449979

[B6] Blom JW Vanderschoot JP Oostindier MJ Osanto S van der Meer FJ Rosendaal FR Incidence of venous thrombosis in a large cohort of 66,329 cancer patients: results of a record linkage study J Thromb Haemost 2006 4 529 535 10.1111/j.1538-7836.2006.01804.x 16460435

[B7] Khorana AA Francis CW Culakova E Fisher RI Kuderer NM Lyman GH Thromboembolism in hospitalized neutropenic cancer patients J Clin Oncol 2006 24 484 490 10.1200/JCO.2005.03.8877 16421425

[B8] Khorana AA Francis CW Culakova E Kuderer NM Lyman GH Frequency, risk factors, and trends for venous thromboembolism among hospitalized cancer patients Cancer 2007 110 2339 2346 10.1002/cncr.23062 17918266

[B9] Khorana AA Kuderer NM Culakova E Lyman GH Francis CW Development and validation of a predictive model for chemotherapy-associated thrombosis Blood 2008 111 4902 4907 10.1182/blood-2007-10-116327 18216292 PMC2384124

[B10] Levitan N Dowlati A Remick SC Tahsildar HI Sivinski LD Beyth R Rates of initial and recurrent thromboembolic disease among patients with malignancy versus those without malignancy. Risk analysis using Medicare claims data Medicine (Baltimore) 1999 78 285 291 10.1097/00005792-199909000-00001 10499070

[B11] Sallah S Wan JY Nguyen NP Venous thrombosis in patients with solid tumors: determination of frequency and characteristics Thromb Haemost 2002 87 575 579 12008937

[B12] Stein PD Beemath A Meyers FA Skaf E Sanchez J Olson RE Incidence of venous thromboembolism in patients hospitalized with cancer Am J Med 2006 119 60 68 10.1016/j.amjmed.2005.06.058 16431186

[B13] Khorana AA Connolly GC Assessing risk of venous thromboembolism in the cancer patient J Clin Oncol 2009 27 29 4839 47 10.1200/JCO.2009.22.3271 19720906 PMC2764392

[B14] Wun T White RH Venous thromboembolism (VTE) in patients with cancer: epidemiology and risk factors Cancer Invest 2009 27 Suppl 1 63 74 19291526 10.1080/07357900802656681

[B15] Blom JW Doggen CJ Osanto S Rosendaal FR Malignancies, prothrombotic mutations, and the risk of venous thrombosis JAMA 2005 293 715 722 10.1001/jama.293.6.715 15701913

[B16] Sousou T Khorana A Identifying cancer patients at risk for venous thromboembolism Hamostaseologie 2009 29 121 124 19151861

[B17] Anderson FA Jr Spencer FA Risk factors for venous thromboembolism Circulation 2003 107 I9 16 10.1161/01.CIR.0000046771.12875.6C 12814980

[B18] Agnelli G Becattini C Venous thromboembolism and atherosclerosis: common denominators or different diseases? J Thromb Haemost 2006 4 1886 1890 10.1111/j.1538-7836.2006.02138.x 16961596

[B19] Prandoni P Bilora F Marchiori A Bernardi E Petrobelli F Lensing AW An association between atherosclerosis and venous thrombosis N Engl J Med 2003 348 1435 1441 10.1056/NEJMoa022157 12686699

[B20] Prandoni P Venous thromboembolism and atherosclerosis: is there a link? J Thromb Haemost 2007 5 Suppl 1 270 275 17635736 10.1111/j.1538-7836.2007.02467.x

[B21] van der Hagen PB Folsom AR Jenny NS Heckbert SR O'Meara ES Reich LM Subclinical atherosclerosis and the risk of future venous thrombosis in the Cardiovascular Health Study J Thromb Haemost 2006 4 1903 1908 10.1111/j.1538-7836.2006.02096.x 16961598

[B22] White RH Chew HK Zhou H Parikh-Patel A Harris D Harvey D Incidence of venous thromboembolism in the year before the diagnosis of cancer in 528,693 adults Arch Intern Med 2005 165 1782 1787 10.1001/archinte.165.15.1782 16087828

[B23] Baron JA Gridley G Weiderpass E Nyren O Linet M Venous thromboembolism and cancer Lancet 1998 351 1077 1080 10.1016/S0140-6736(97)10018-6 9660575

[B24] Sorensen HT Mellemkjaer L Steffensen FH Olsen JH Nielsen GL The risk of a diagnosis of cancer after primary deep venous thrombosis or pulmonary embolism N Engl J Med 1998 338 1169 1173 10.1056/NEJM199804233381701 9554856

[B25] Kessler CM The link between cancer and venous thromboembolism: a review Am J Clin Oncol 2009 32 S3 S7 10.1097/COC.0b013e3181b01b17 19654481

